# Molecular Data Reveal Multiple Lineages in Piranhas of the Genus *Pygocentrus* (Teleostei, Characiformes)

**DOI:** 10.3390/genes10050371

**Published:** 2019-05-15

**Authors:** Nadayca T.B. Mateussi, Bruno F. Melo, Fausto Foresti, Claudio Oliveira

**Affiliations:** Departamento de Morfologia, Instituto de Biociências, Universidade Estadual Paulista, Rua Professor Doutor Antônio Celso Wagner Zanin, 250, Rubião Junior, Botucatu, SP 18618-689, Brazil; brunfmelo@gmail.com (B.F.M.); f.foresti@unesp.br (F.F.); claudio@ibb.unesp.br (C.O.)

**Keywords:** biodiversity, DNA barcode, Neotropical region, Serrasalmidae

## Abstract

Carnivorous piranhas are distributed in four serrasalmid genera including *Pygocentrus,* which inhabit major river basins of South America. While *P. cariba* and *P. piraya* are endemics of the Orinoco and São Francisco basins, respectively, *P. nattereri* is widely distributed across the Amazonas, Essequibo, lower Paraná, Paraguay, and coastal rivers of northeastern Brazil, with recent records of introductions in Asia. Few studies have focused on the genetic diversity and systematics of *Pygocentrus* and the putative presence of additional species within *P. nattereri* has never been the subject of a detailed molecular study. Here we aimed to delimit species of *Pygocentrus*, test the phylogeographic structure of *P. nattereri*, and access the origin of introduced specimens of *P. nattereri* in Asia. Phylogenetic analyses based on a mitochondrial dataset involving maximum-likelihood tree reconstruction, genetic distances, Bayesian analysis, three delimitation approaches, and haplotype analysis corroborate the morphological hypothesis of the occurrence of three species of *Pygocentrus*. However, we provide here strong evidence that *P. nattereri* contains at least five phylogeographically-structured lineages in the Amazonas, Guaporé (type locality), Itapecuru, Paraná/Paraguay, and Tocantins/Araguaia river basins. We finally found that the introduced specimens in Asia consistently descend from the lineage of *P. nattereri* from the main Rio Amazonas. These results contribute to future research aimed to detect morphological variation that may occur in those genetic lineages of *Pygocentrus*.

## 1. Introduction

The Neotropical fish family Serrasalmidae contains 16 genera and 97 valid species [1] of ecomorphologically diverse freshwater fishes popularly known as pacus and piranhas. The species are divided in three main clades being two encompassed by pacus, tambaquis and silverdollars and a third containing mostly carnivorous piranhas [2]. This later clade includes six genera: the four carnivorous genera *Pristobrycon* Eigenmann, 1915, *Pygocentrus* Müller & Troschel, 1844, *Pygopristis* Müller & Troschel, 1844, and *Serrasalmus* Lacepède, 1803, the lepidophagous genus *Catoprion* Müller & Troschel, 1844 and the omnivorous *Metynnis* Cope 1878 [2,3,4]. The monophyly of this six-genera group is supported on the basis of both morphological [3,4] and multilocus molecular data [2,5,6].

The genus *Pygocentrus* includes the largest species of piranhas, reaching up to 50 cm standard length [7], that are highly appreciated in the ornamental trade and have a relative economic importance in regional fisheries and aquaculture [8,9]. Species of *Pygocentrus* are morphologically distinguished from other serrasalmids by a substantially wider head, a dorsal profile that is moderately to strongly convex, the presence of a preanal spine that is often undetectable externally, tricuspid teeth and the lack of ectopterygoid teeth, except in small juveniles [10,11]. The main synapomorphy of the genus is the presence of crests around the lateral-sensorial system of the frontal, parietal and pterotic bones [12]. The genus is monophyletic and hypothesized to be the sister clade of *Serrasalmus* plus *Pristobrycon calmoni* [2,13].

**Figure 1:**
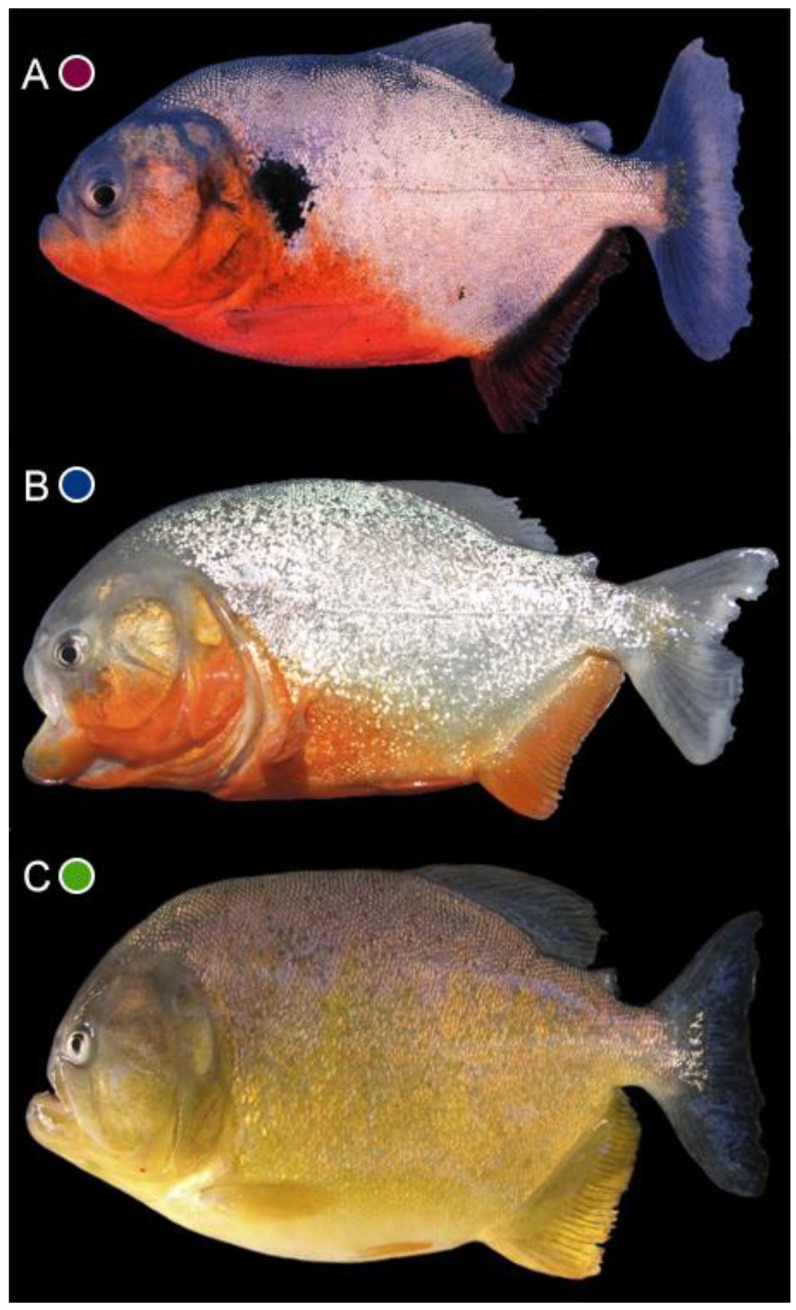
Representative specimens of *Pygocentrus*: (**A**) *P. cariba*, Río Apure, LBP 10225. (**B**) *P. nattereri*, Rio das Mortes, Araguaia basin. (**C**) *P. piraya*, Rio São Francisco. Photographs by Alec Zeinad (**B**,**C**), specimens not preserved.

The taxonomic revision of *Pygocentrus* [10] recognized three species: *P. cariba* (Humboldt, 1821), an endemic from the Río Orinoco basin; *P. nattereri* Kner, 1858, widely-distributed in the Amazonas, Guianas, lower Paraná, Paraguay, and coastal rivers of northeastern Brazil; and *P. piraya* (Cuvier, 1819), the type-species of the genus, endemic from the Rio São Francisco basin (Figure 1). Fink [10] and Fink and Zelditch [14] did not find morphological or morphometric evidence that would support additional species within *P. nattereri*, leaving two nominal species in synonymy of *P. nattereri*: *P. altus* Gill, 1871, from the Río Marañon, upper Rio Amazonas, and *P. ternetzi* (Steindachner, 1908), from the Rio Paraguay. The three-species hypothesis were only recently tested in a broader barcoding study of the entire family Serrasalmidae that recognized both *P. cariba* and *P. piraya* but presented variable numbers of entities within *P. nattereri* depending on the delimitation analyses [15]. These analytical inconsistencies and the limited taxon sampling from relatively few Amazonian regions indicate the necessity of an intrageneric analysis to refine the species delimitation analyses, including samples from multiple South American river basins.

The variable number of species delimited for the red-bellied piranha *Pygocentrus nattereri* [15] is hypothesized by the genetic structure of lineages from distinct river systems. For example, the phylogeographic study of *Pygocentrus* based on the mtDNA control region found *P. nattereri* with structured genetic lineages in which the Paraná, Ucayali and Madeira lineages appeared genetically closer to each other than to the lineage from mainstream Rio Amazonas [13]. Population genetic studies within *P. nattereri* from the northeastern Brazil [16] and from the Rio Solimões/Amazonas [17] have also shown high levels of genetic diversity and significant genetic differentiation among populations. The maintenance of *P. nattereri* in captivity likely enabled the introduction of *P. nattereri* in several Asian rivers including in Bangladesh [18], China [9] and the Philippines [19,20]. However, studies reporting introductions lack evidence for the precise geographic origin of those parental specimens in South America.

Here, we used partial sequences of the mitochondrial gene *cytochrome c oxidase subunit I* (*COI*) and modern phylogenetic and species delimitation methods in order to (1) test the morphological hypothesis of the presence of three species of *Pygocentrus* [10], (2) test the population genetic hypothesis of multiple genetically-structured populations of *P. nattereri* [13,16,17], and (3) determine the geographic origin of recently introduced specimens of *P. nattereri* in Asia [20].

## 2. Materials and Methods

### 2.1. Taxon Sampling

**Figure 2:**
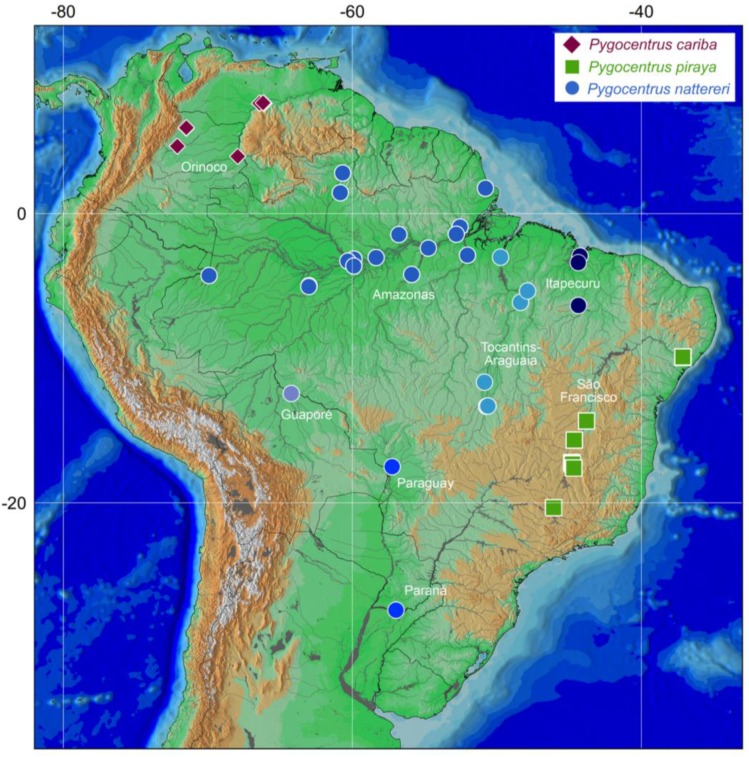
Distribution map of specimens of *Pygocentrus*. Different shades of blue represent distinct genetic lineages of *P. nattereri* found in this study.

Specimens were collected or obtained from fish collections, and morphologically identified by consulting the taxonomic literature and identification keys [10]. Specimens of the three valid species of *Pygocentrus* plus *Serrasalmus elongatus* Kner, 1858 as outgroup (root) were included in the analysis (Appendix A; Figure 2). The matrix contained 161 specimens, in which 57 were newly sequenced and 104 were obtained from GenBank (ncbi.nlm.nih.gov/genbank) or BOLD (boldsystems.org) databases (Appendix A). We attempted to obtain samples from all river basins in order to sample intraspecific genetic diversity for each species. We also included available sequences of *P. nattereri* introduced in the Philippines [20], the only available sequences on GenBank, to identify the original region that served as the source of those introduced specimens. Vouchers were fixed in 95% ethanol or 10% formalin and transferred to 70% ethanol for permanent storage and posteriorly deposited in the Laboratório de Biologia e Genética de Peixes, Universidade Estadual Paulista, Botucatu, Brazil (LBP), and Colección de Zoología, Universidad del Tolima, Ibagué, Colombia (CZUT-IC) (Appendix A).

### 2.2. DNA Extraction, Amplification and Sequencing

Tissue samples were taken from livers, gills, fins or muscles. The total DNA was isolated using the Qiagen “DNeasy Blood & Tissue” (Qiagen, Hilden, Germany) kit according to manufacturer’s instructions. Partial segments of the *COI* gene were amplified by PCR using the primers Fish F1 (5′-TCAACCAACCACAAAGACATTGGCAC-3′) and Fish R1 (5′–TAGACTTCTGGGTGGCCAAAGAATCA–3′) [21]. The PCR was performed on a thermocycler with a final volume of 12 µL containing of 8.175 µL distilled water, 0.5 µL dNTP (8 mM), 1.25 µL 10× Taq buffer (500 mM KCl; 200 mM Tris-HCl), 0.375 µL of MgCl_2_, 0.25 µL of each primer (10 µM) and 0.2 µL of PHT Taq polymerase. PCR conditions consisted of an initial denaturation at 95 °C for 5 min, followed by 35 cycles including denaturation at 95 °C for 45 s, annealing at 52 °C for 45 s and extension at 68 °C for 120 s, and a final extension at 68 °C for 5 min. Amplified products were checked on 1% agarose gel.

Amplicons were then purified with ExoSAP-IT (USB Corporation, Cleveland, OH, USA) following the manufacturer’s protocol. The purified product was used as template to sequence both DNA strands. The cycle sequencing reaction was carried out using a BigDye Terminator v3.1 Cycle Sequencing Ready Reaction kit (Applied Biosystems, Austin, TX, USA) in a final volume of 7 µL containing 0.35 µL primer (10 mM), 1.05 µL buffer 5×, 0.7 µL BigDye mix, and 3.9 µL distilled water. The cycle sequencing conditions were initial denaturation at 96 °C for 2 min followed by 30 cycles of denaturation at 96 °C for 45 s, annealing at 50 °C for 60 s, and extension at 60 °C for 4 min. The sequencing products were then purified following the protocol suggested in the BigDye Terminator v3.1 Cycle Sequencing kit’s manual (Applied Biosystems). All samples were sequenced on an ABI 3130 Genetic Analyzer (Applied Biosystems) following the manufacturer’s instructions.

### 2.3. Species Delimitation Analyses

Sequences were assembled and edited in Geneious 4.8 [22] to obtain a single consensus sequence for each specimen and also to check for deletions, insertions, and stop codons. Then, sequences were aligned with Muscle algorithm [23], and the aligned matrix was tested for saturation in DAMBE v7 [24]. The TN93+I (Tamura-Nei + Invariant sites) was estimated as the best-fit model of nucleotide evolution for our data by PartitionFinder [25] and was used in programs containing such a model. Sequences were binned into groups according to a neighbor-joining tree using TN93 in MEGA X [26]; for example, subgroups of *Pygocentrus nattereri* were split in five drainage-groups (Amazonas, Guaporé, Itapecuru, Paraná-Paraguay, and Tocantins/Araguaia) to test the prior hypothesis of multiple structured populations. The Amazonas population includes samples from the entire basin, except for Guaporé and Tocantins-Araguaia river basins as determined by the distance analysis. Three approaches of genetic distances were obtained using the TN93 model in MEGA X: the overall mean distance, intraspecific distances, and interspecific distances. The neighbor-joining tree was then generated in MEGA and tested by 1000 bootstrap pseudoreplicates.

We used three distinct species delimitation methods (Poisson Tree Process, Automatic Barcode Gap Discovery, and General Mixed Yule Coalescent Model) for our dataset using either sequence-based estimations or topology-based analyses based on the maximum likelihood (ML) or Bayesian inference. The maximum likelihood (ML) analysis was performed in RAxML v7.2 [27] using the GTR-GAMMA model, a maximum parsimony starting tree, and a posteriori analysis of bootstrap with the autoMRE function [28]. The best ML tree was used as an input tree for the Poisson Tree Process (PTP) model, that delimits species using non-ultrametric trees, since the speciation rate is modeled directly by the number of nucleotide substitutions [29]. The analysis was performed with the PTP webserver (species.h-its.org/ptp) using 100,000 MCMC generations and a 0.1 burn-in rate as the default settings.

Secondly, we performed the Automatic Barcode Gap Discovery (ABGD) analysis, an automatic procedure that sorts sequences into hypothetical species based on the barcode gap [30]. It infers a model-based confidence limit for intraspecific divergence by detecting the barcode gap as the first significant gap beyond this limit and uses it to partition the data. Inference of the limit and gap detection are then recursively applied to previously obtained groups to get finer partitions until there is no further partitioning [30]. The analysis was performed at the ABGD webserver (wwwabi.snv.jussieu.fr/public/abgd/abgdweb.html) with the Kimura (K80; 2.0) distance model with X = 1.0, Pmin = 0.001 and Pmax = 0.05.

Finally, we ran the General Mixed Yule Coalescent model (GMYC), a likelihood method that delimits species by fitting within- and between species branching models to reconstructed gene trees [31]. Because GMYC requires no polytomies, DAMBE v7 [24] was used to remove duplicated haplotypes, which improves the algorithm and maximizes computational time analysis. Then, a Bayesian inference of phylogeny was estimated with a relaxed lognormal clock with a speciation birth-death model, on an arbitrary timescale, using BEAST v1.8.4 [32]. The nucleotide evolution model used to estimate the ultrametric tree was TN93+I as estimated by PartitionFinder [25]. A random tree was used as a starting tree for the MCMC searches with two independent runs of 500,000,000 generations, with trees sampled at every 50,000th generation. The distribution of log-likelihood scores was examined to determine the stationary phase for each search and to decide whether extra runs were required to achieve convergence using Tracer v1.7.1 [33]. All sampled topologies beneath the asymptote were discarded as part of a burn-in procedure (10%), and the remaining trees were used to construct a 50% majority-rule consensus tree in TreeAnnotator v1.8.4. The resulting tree was visualized in FigTree v1.4.3, and the resultant topology was implemented in the GMYC analysis. The GMYC delimitation analysis was performed at the webserver (species.h-its.org/gmyc) with a single threshold method and other parameters set as default.

We also used DnaSP v5 [34] to estimate the number of polymorphic sites, haplotype number and haplotype/nucleotide diversity (H_D_/Pi) and used PopART v1.7 [35] to run a median-joining analysis [36] and obtain a haplotype network. Finally, we used a PhyloMap-PTP tool [37] available in the PTP webserver that combines Principal Coordinates Analysis (PCoA), PTP, and species tree mapping. These approaches were applied to understand the spatial distribution of haplotypes and how they are related to each other.

## 3. Results

Newly generated sequences were obtained from 57 specimens in addition to 104 sequences obtained from public databases, resulting in a final matrix with 161 sequences. Sequences are deposited in BOLD PYGO001-18–048-18 and PYGO049-19–057-19. Stop codons, deletions or insertions were absent in all sequences. Following alignment and editing, the final matrix has 522 bp of which 476 bp were conserved (91.2%) and 46 were variable, with 22.6% adenine, 31.8% cytosine, 27.9% thymine and 17.8% guanine. DAMBE revealed Iss values lower than Iss.cAsym and Iss.cSym values, which mean the lack of a saturation signal in the matrix. The dataset contains a total of 12 haplotypes (Pi = 12.157; H_D_ = 0.835): one haplotype of *Serrasalmus* as root and 11 haplotypes of *Pygocentrus*. *Pygocentrus cariba* presented two haplotypes, *P. piraya* presented four haplotypes, and *P. nattereri* presented six haplotypes. Within *P. nattereri*, each sample from Amazonas, Guaporé, Itapecuru, Paraná/Paraguay and Tocantins/Araguaia presented exclusive haplotypes.

The genetic distance analysis recognizes the three morphologically-defined species of *Pygocentrus* with 0.059 ± 0.010 of distance between *P. cariba* and *P. piraya*, 0.055 ± 0.010 between *P. cariba* and *P. nattereri*, and 0.026 ± 0.006 between *P. piraya* and *P. nattereri*. Subgroups of *P. nattereri* presented genetic distances ranging from 0.005 ± 0.003 between Guaporé and Paraná/Paraguay to 0.017 ± 0.005 between Itapecuru and Tocantins/Araguaia and Itapecuru and Guaporé (Table 1). Results also reveal low intraspecific genetic variation within each lineage (0.000–0.003) (Table 1).

**Figure 3:**
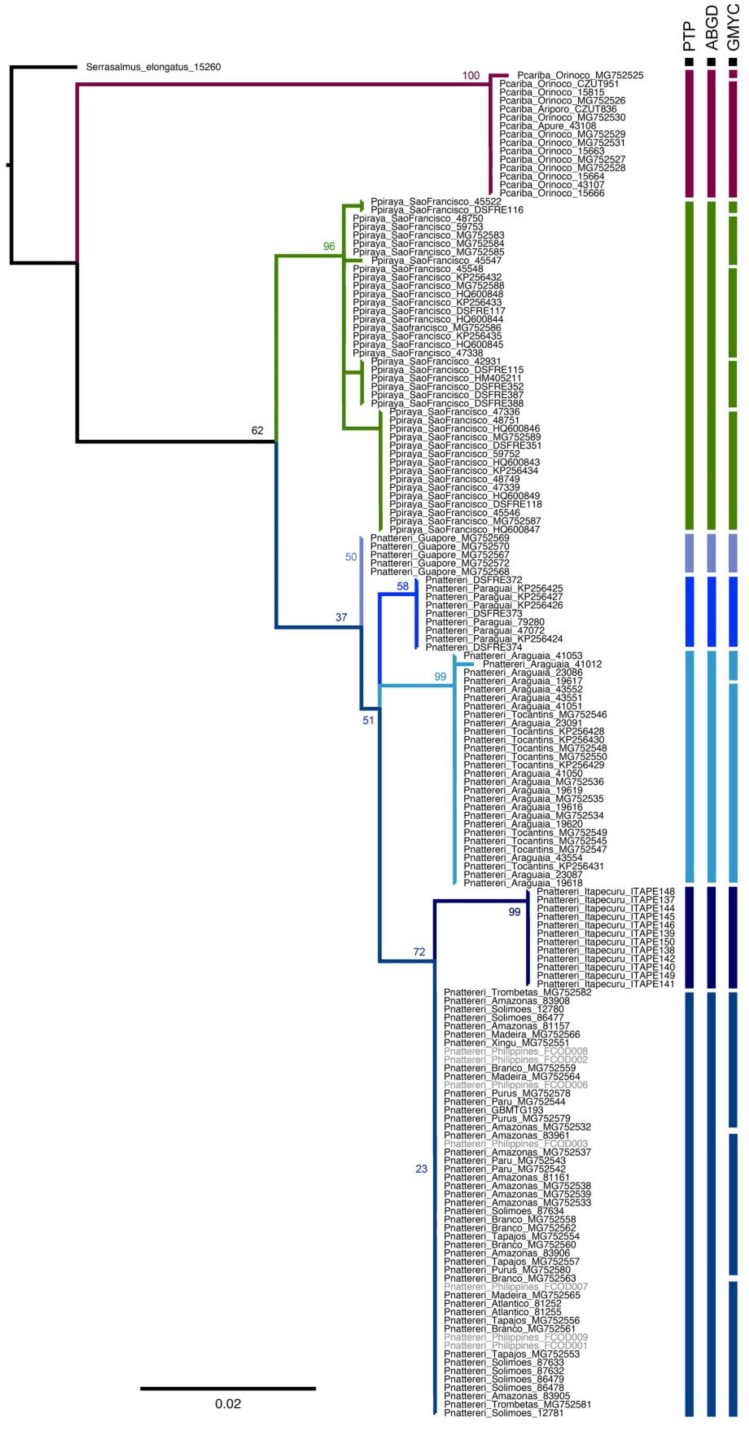
Best maximum likelihood tree based on the *cytochrome c oxidase subunit I* gene for *Pygocentrus* species evidencing the presence of multiple genetic lineages within *P. nattereri*. Colored bars after tip names represents results of the three species delimitation analyses. GMYC results for the Amazonas lineage of *P. nattereri* are not delimited by taxa. Numbers near nodes indicate bootstrap support.

All topologies returned very similar results regarding the position of each lineage. Neighbor-joining (Figure S1), ML (Figure 3 and Figure S2) and the Bayesian tree (Figure S3) recognized each of the three previously recognized species of *Pygocentrus* and also indicates a clear segmentation of lineages in *P. nattereri* (Figure 3). The PTP method returned well-defined lineages for *P. cariba* and *P. piraya* and splitted *P. nattereri* in five distinct lineages from Amazonas, Guaporé, Itapecuru, Paraná/Paraguay, and Tocantins/Araguaia. The ABGD method resulted in eight partitions that ranged from 11 (*p* = 0.001) to two lineages (*p* = 0.02), with three partitions supporting the presence of seven lineages of *Pygocentrus* (*p* = 0.002–0.005), that is *P. cariba*, *P. piraya,* and *P. nattereri* subdivided in five subgroups: Amazonas, Guaporé, Itapecuru, Paraná/Paraguay and Tocantins/Araguaia. The GMYC oversplitted *Pygocentrus* in 16 lineages, two for *P. cariba*, five for *P. piraya* and eight for *P. nattereri* (three in the Amazonas, two in the Tocantins/Araguaia, and one for each Guaporé, Itapecuru, and Paraná/Paraguay). The threshold time obtained in the GMYC analysis was −1.14 × 10^−4^T, where T is the time from present to the time of the root.

**Figure 4:**
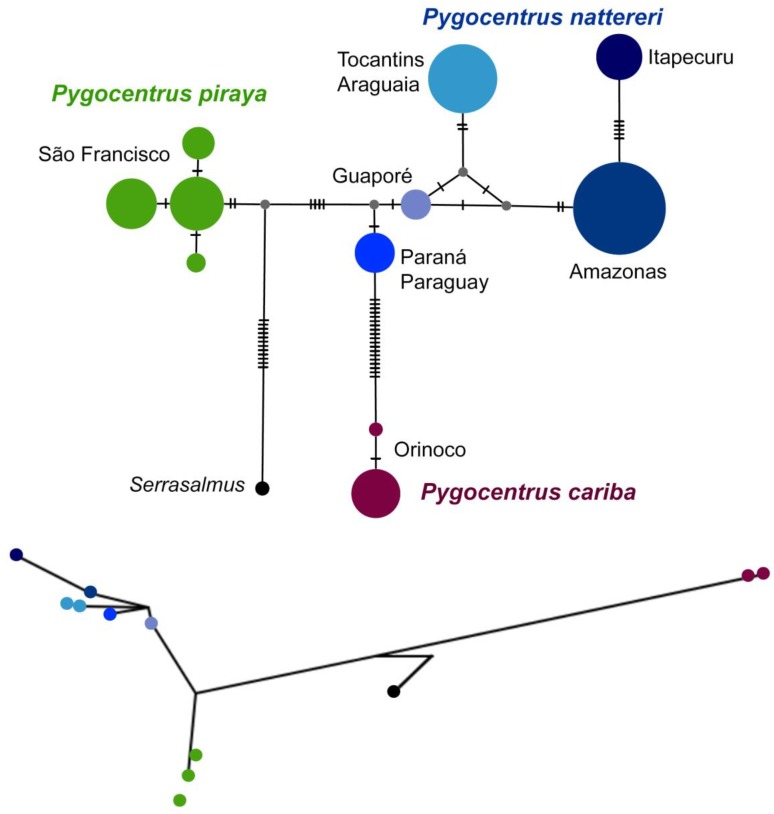
Haplotype network (above) and PhyloMap-PTP (below) showing the distribution of the 11 distinct haplotypes of *Pygocentrus*.

Additionally, we included seven sequences of introduced specimens of *Pygocentrus nattereri* in the Philippines [20] to determine the source of parental specimens that were originally from South America. All topologies evidenced that they are genetically proximate to the Amazonas group (Figures S1 and S2). The sequences of specimens from Philippines (FCOD numbers) do not have any nucleotide substitution when compared to those collected in the Amazonas drainages (i.e., 0.000 genetic distance). This evidence indicates that the introduced specimens were obtained from somewhere in the Amazonas basin other than in the Guaporé or Tocantins/Araguaia or any other South American drainage. Haplotype network and PhyloMap-PTP approaches allow the visualization of the distribution and relationships of each haplotype (Figure 4).

## 4. Discussion

Species delimitation results support the recognition of the two species *Pygocentrus cariba* (Río Orinoco) and *P. piraya* (Rio São Francisco), and reveal the presence of five genetic lineages within the widely distributed *P. nattereri*. The three methods (PTP, ABGD and GMYC) split *P. nattereri* into five lineages: Amazonas, Guaporé, Itapecuru, Paraná/Paraguay, and Tocantins/Araguaia, and with GMYC splitting *P. cariba* and *P. piraya* in two and five entities in the Orinoco and São Francisco basins, respectively. After the examination of voucher specimens using traditional morphometric/meristic data for Serrasalmidae [38], we did not identify morphological variation or diagnoses to formally describe these genetic lineages (or potential species). Thus, we recognize the three current species of *Pygocentrus* and the presence of five structured populations of *P. nattereri* in South America. These lineages can be potentially sibling species *sensu* Mayr [39], representing the herein named *P. nattereri* species complex. Sibling species represent a special case of cryptic species, when they are closest relatives and are not distinguished from one another, taxonomically [39,40]. Similarly, recent studies have been revealed several examples of cryptic species in Neotropical freshwater fish, mostly due to advances in molecular systematics and integrative taxonomy [41,42,43].

*Pygocentrus nattereri* is the most abundant and widely distributed species of *Pygocentrus* and, accordingly, has controversial species boundaries and carries a history of doubts about its diagnostic features, validity and taxonomic status. Fink [10] performed a revision of *Pygocentrus* and could not find any exclusive character supporting its species status, despite analyzing *P. nattereri* from all drainages. However, he delimited *P. nattereri* by the combination of characters such as absence of humeral blotch in adults and lack of rays in the adipose fin. Type specimens of *P. nattereri* were assigned to rio Guaporé of the rio Madeira basin [10] and two names currently in synonym of *P. nattereri* are available for *Pygocentrus*: *P. altus* from the upper Rio Amazonas that could be applied for the Amazonas lineage, and *P. ternetzi* from the Rio Paraguay that could be applied for the Paraná/Paraguay lineage. However, we consider prematurely revalidating those species without a taxonomic revision, taking into account our strong molecular evidence for the occurrence of additional lineages within the present concept of *P. nattereri*.

Our results agree with the most recent barcoding study of the family Serrasalmidae that included all species of *Pygocentrus* [15] and recognized both *P. cariba* and *P. piraya* as two species, with segmentation of *P. nattereri* in multiple lineages depending on the delimitation approach. The authors [15] found two well-defined lineages of *P. nattereri* (Tocantins/Araguaia lineage, and Branco/Madeira/Tapajós lineage) with GMYC recognizing a third lineage from the Rio Guaporé (Madeira basin). Our results indicate those same clusters and added two additional ones: the Itapecuru and Paraná/Paraguay lineages (Figure 3). Present data also support the previous phylogeographic hypothesis that *P. nattereri* contains structured populations along the wide continental distribution [10,13] and also delimit each genetic lineage along the distribution of the species. It is noteworthy that additional samples from Guianas and other remote regions of Amazonia can be added to our dataset to further delimit *P. nattereri*.

Results presented herein indicate a very low genetic variation among most species of *Pygocentrus*, evident in *P. piraya* and within the *P. nattereri* complex, as exemplified by the low genetic distance values (Table 1) and the presence of few haplotypes even including species from a broad geographic expanse (Figure 4). For example, we identified an exclusive haplotype that is shared between specimens of *P. nattereri* collected in the Rio Solimões at Brazil/Colombia boundary and from Amapá lakes at the northern Amazonas estuary. *Pygocentrus cariba* presents the highest genetic distance values among *Pygocentrus* species, even more than *S. elongatus* with other *Pygocentrus*. In fact, Hubert et al. [13] found a rapid speciation between *P. cariba* and the ancestor of *P. nattereri* and *P. piraya* less than one million year after the split between *Pygocentrus* and *Serrasalmus* (~8.73 Ma vs. 8.0 Ma). On the other hand, the cladogenetic events leading to *P. nattereri* and *P. piraya* were much more recent at around 2.63 ± 0.2 Ma, the split of *P. nattereri* from the Amazonas and that from the upper Paraguay at around 1.8 Ma and that from the Paraná at about 1.77 ± 0.3 Ma, and the differentiation of the lineages from the upper Amazonas (Ucayali and Madeira) at around 0.79 ± 0.1 Ma, which suggest a rapid and relatively recent differentiation of *P. nattereri* and *P. piraya* lineages. Accordingly, Machado et al. [15] found *P. cariba* to be the first species to diverge from any other species of *Pygocentrus* or *Serrasalmus*.

Species of *Pygocentrus* are widely introduced outside their native ranges and the environment impacts are specially related to predation of native species and damage of fishing nets and other fishes [44,45]. Herein, sequences of *Pygocentrus* introduced in the Philippines [20] were included in the analyses and results indicate that they belong to the Amazonas lineage. The effects of an invasion can be both observed on single or small groups of species or through an entire ecosystem; impacts such as predation, herbivory, parasitism, disease, competition and hybridization led to extirpation or reduction of the local population, or even causing global extinction of native species [46]. The recognition of the invasive species is the first step towards the investigation and management actions that may follow, such as eradication, maintenance management and control of population density [46]. Since the effects of introduction of these specimens may lead to ecological damage (e.g., competition for food, space and spawning sites), the accurate information about origin of introduced specimens of *P. nattereri* might contribute for future local management purposes.

Morphological characters are traditionally used to discriminate Serrasalmidae species despite allometry and body coloration being highly variable during ontogeny, thus strongly affecting accurate species identifications [10]. The combination of morphological and molecular approaches appears to be a good point to study interspecific variation and, indeed, has helped to identify, discriminate and describe species of other serrasalmid genera. For example, the study including three recognized species of *Mylossoma* indicated five genetic lineages instead [47], with two species resurrected and redescribed afterwards (*M. albiscopum* and *M. unimaculatum*; [48]). In a similar vein, Andrade et al. [49] recognized the seventh species of *Tometes* by integrating both morphological and mitochondrial data. The results presented herein integrate these two studies and expand the promising field of integrative taxonomy of Serrasalmidae. Together, these studies indicate the need for deep revisions of species and genera of Serrasalmidae, involving both genetic and morphological data to determine the presence of potential undescribed species and to reassign species among genera. In this context, further research can address additional morphological characters in order to test our molecular hypothesis of the presence of seven genetic lineages of *Pygocentrus* in South America that can be potentially be recognized as valid species.

## Figures and Tables

**Table 1:** Pairwise TN93 genetic distance values among drainage-based lineages of *Pygocentrus*. Bold numbers represent intraspecific genetic variation. Amaz = Amazonas; Itapec = Itapecuru; Toc/Ara = Tocantins/Araguaia; Par/Par = Paraná/Paraguay; Guap = Guaporé.

	*P. cariba*	*P. piraya*	*P. nattereri* Amaz	*P. nattereri* Itapec	*P. nattereri* Toc*/*Ara	*P. nattereri* Par*/*Par	*P. nattereri* Guap	*S. elongatus*
*P. cariba*	**0.000 ± 0.000**							
*P. piraya*	0.059 ± 0.010	**0.003 ± 0.001**						
*P. nattereri* Amaz	0.051 ± 0.010	0.025 ± 0.006	**0.000 ± 0.000**					
*P. nattereri* Itapec	0.056 ± 0.010	0.035 ± 0.008	0.009 ± 0.004	**0.000 ± 0.000**				
*P. nattereri* Toc*/*Ara	0.056 ± 0.010	0.027 ± 0.007	0.011 ± 0.004	0.017 ± 0.005	**0.000 ± 0.000**			
*P. nattereri* Par*/*Par	0.045 ± 0.009	0.019 ± 0.005	0.009 ± 0.004	0.015 ± 0.005	0.011 ± 0.004	**0.000 ± 0.000**		
*P. nattereri* Guap	0.050 ± 0.009	0.017 ± 0.005	0.007 ± 0.003	0.017 ± 0.005	0.009 ± 0.039	0.005 ± 0.003	**0.000 ± 0.000**	
*S. elongatus*	0.046 ± 0.009	0.037 ± 0.008	0.041 ± 0.009	0.048 ± 0.009	0.042 ± 0.009	0.037 ± 0.008	0.040 ± 0.008	**-**

## Supplementary Materials

The following are available online at https://www.mdpi.com/2073-4425/10/5/371/s1, Figure S1: Neighbor joining tree based on the *cytochrome c oxidase I* gene for *Pygocentrus* species. Figure S2: Maximum likelihood tree based on the *cytochrome c oxidase I* gene for *Pygocentrus* species Figure S3: Bayesian inference tree based on the *cytochrome c oxidase I* gene for *Pygocentrus* species.

## Appendix A

**Table A1:** Voucher, locality information and BOLD or Genbank accession numbers of the analyzed specimens of *Pygocentrus*.

Haplotype	Taxon	Voucher	Specimen	Locality, Basin	City, State	Country	Accession n.
1	*P. cariba*	LBP 2229	15663	Río Orinoco basin	Caicara del Orinoco, Bolívar	Venezuela	PYGO003-18
1	*P. cariba*	LBP 2229	15664	Río Orinoco basin	Caicara del Orinoco, Bolívar	Venezuela	PYGO004-18
1	*P. cariba*	LBP 2229	15666	Río Orinoco basin	Caicara del Orinoco, Bolívar	Venezuela	PYGO005-18
1	*P. cariba*	LBP 2290	15815	Río Orinoco basin	Caicara del Orinoco, Bolívar	Venezuela	PYGO006-18
1	*P. cariba*	LBP 10225	43107	Río Apure, Orinoco basin	Cabruta, Guarico	Venezuela	PYGO001-18
1	*P. cariba*	LBP 10225	43108	Río Apure, Orinoco basin	Cabruta, Guarico	Venezuela	PYGO002-18
1	*P. cariba*	CZUT-IC 12810	836	Río Ariporo, Orinoco basin	-	Colombia	PYGO054-19
1	*P. cariba*	CZUT-IC 11395	951	Caño Materro, Orinoco basin	-	Colombia	PYGO052-19
2	*P. cariba*	UFAM 13525	13525	Río Orinoco basin	Guainía	Colombia	MG752525
1	*P. cariba*	UFAM 13526	13526	Río Orinoco basin	Guainía	Colombia	MG752526
1	*P. cariba*	UFAM 13529	13529	Río Orinoco basin	Guainía	Colombia	MG752527
1	*P. cariba*	UFAM 13741	13741	Río Orinoco basin	Guainía	Colombia	MG752528
1	*P. cariba*	UFAM 13743	13743	Río Orinoco basin	Guainía	Colombia	MG752529
1	*P. cariba*	UFAM 13744	13744	Río Orinoco basin	Guainía	Colombia	MG752530
1	*P. cariba*	UFAM 13745	13745	Río Orinoco basin	Guainía	Colombia	MG752531
6	*P. nattereri*	INPA 41663	102036	Rio Purus, Amazon basin	Amazonas	Brazil	MG752578
6	*P. nattereri*	INPA 41689	102084	Rio Purus, Amazon basin	Amazonas	Brazil	MG752579
6	*P. nattereri*	INPA 41689	102085	Rio Purus, Amazon basin	Amazonas	Brazil	MG752580
6	*P. nattereri*	INPA 50418	105742	Rio Trombetas, Amazon basin	Pará	Brazil	MG752581
6	*P. nattereri*	INPA 50175	105817	Rio Trombetas, Amazon basin	Pará	Brazil	MG752582
6	*P. nattereri*	LBP 1697	12780	Lago do Vanico, Amazon basin	Carero, Amazonas	Brazil	PYGO031-18
6	*P. nattereri*	LBP 1697	12781	Lago do Vanico, Amazon basin	Carero, Amazonas	Brazil	PYGO032-18
9	*P. nattereri*	LBP 2978	19616	Rio Araguaia, Amazon basin	Cocalinho, Mato Grosso	Brazil	PYGO014-18
9	*P. nattereri*	LBP 2978	19617	Rio Araguaia, Amazon basin	Cocalinho, Mato Grosso	Brazil	PYGO015-18
9	*P. nattereri*	LBP 2978	19618	Rio Araguaia, Amazon basin	Cocalinho, Mato Grosso	Brazil	PYGO016-18
9	*P. nattereri*	LBP 2978	19619	Rio Araguaia, Amazon basin	Cocalinho, Mato Grosso	Brazil	PYGO017-8
9	*P. nattereri*	LBP 2978	19620	Rio Araguaia, Amazon basin	Cocalinho, Mato Grosso	Brazil	PYGO018-18
9	*P. nattereri*	LBP 4000	23086	Rio Araguaia, Amazon basin	S. Félix Araguaia, Mato Grosso	Brazil	PYGO011-18
9	*P. nattereri*	LBP 4000	23087	Rio Araguaia, Amazon basin	S. Félix Araguaia, Mato Grosso	Brazil	PYGO012-18
9	*P. nattereri*	LBP 4000	23091	Rio Araguaia, Amazon basin	S. Félix Araguaia, Mato Grosso	Brazil	PYGO013-18
10	*P. nattereri*	LBP 12641	47072	Rio Cuiabá, Paraguay basin	Corumbá, Mato Grosso do Sul	Brazil	PYGO022-18
9	*P. nattereri*	LBP 12693	43551	Rio Araguaia, Amazon basin	Cocalinho, Mato Grosso	Brazil	PYGO019-18
9	*P. nattereri*	LBP 12693	43552	Rio Araguaia, Amazon basin	Cocalinho, Mato Grosso	Brazil	PYGO020-18
9	*P. nattereri*	LBP 12693	43554	Rio Araguaia, Amazon basin	Cocalinho, Mato Grosso	Brazil	PYGO021-18
9	*P. nattereri*	LBP 12738	41012	Rio Araguaia, Amazon basin	Cocalinho, Mato Grosso	Brazil	PYGO007-18
9	*P. nattereri*	LBP 12738	41050	Rio Araguaia, Amazon basin	Cocalinho, Mato Grosso	Brazil	PYGO008-18
9	*P. nattereri*	LBP 12738	41051	Rio Araguaia, Amazon basin	Cocalinho, Mato Grosso	Brazil	PYGO009-18
9	*P. nattereri*	LBP 12738	41053	Rio Araguaia, Amazon basin	Cocalinho, Mato Grosso	Brazil	PYGO010-18
10	*P. nattereri*	LBP 19961	79280	Rio Paraná, lower Paraná basin	Coratei	Paraguay	PYGO023-18
6	*P. nattereri*	LBP 21836	83905	Rio Negro, Amazon basin	Manaus, Amazonas	Brazil	PYGO024-18
6	*P. nattereri*	LBP 21836	83906	Rio Negro, Amazon basin	Manaus, Amazonas	Brazil	PYGO025-18
6	*P. nattereri*	LBP 21836	83908	Rio Negro, Amazon basin	Manaus, Amazonas	Brazil	PYGO026-18
6	*P. nattereri*	LBP 21836	83961	Rio Negro, Amazon basin	Manaus, Amazonas	Brazil	PYGO027-18
6	*P. nattereri*	LBP 22328	86477	Rio Solimões, Amazon basin	Tabatinga, Amazonas	Brazil	PYGO028-18
6	*P. nattereri*	LBP 22328	86478	Rio Solimões, Amazon basin	Tabatinga, Amazonas	Brazil	PYGO029-18
6	*P. nattereri*	LBP 22328	86479	Rio Solimões, Amazon basin	Tabatinga, Amazonas	Brazil	PYGO030-18
6	*P. nattereri*	LBP 20651	81252	Lago Pracuúba, Atlantic drainage	Pracuúba, Amapá	Brazil	PYGO033-18
6	*P. nattereri*	LBP 20651	81255	Lago Pracuúba, Atlantic drainage	Pracuúba, Amapá	Brazil	PYGO034-18
6	*P. nattereri*	LBP 20977	81157	Rio Jari, Amazon basin	Almeirim, Pará	Brazil	PYGO035-18
6	*P. nattereri*	LBP 20977	81161	Rio Jari, Amazon basin	Almeirim, Pará	Brazil	PYGO036-18
6	*P. nattereri*	LBP 22815	87632	Rio Solimões, Amazon basin	Iranduba, Amazonas	Brazil	PYGO051-19
6	*P. nattereri*	LBP 22815	87633	Rio Solimões, Amazon basin	Iranduba, Amazonas	Brazil	PYGO050-19
6	*P. nattereri*	LBP 22815	87634	Rio Solimões, Amazon basin	Iranduba, Amazonas	Brazil	PYGO049-19
10	*P. nattereri*	NtrMS01	-	Rio Paraguay basin	Unknown	Brazil	KP256424
10	*P. nattereri*	NtrMS02	-	Rio Paraguay basin	Unknown	Brazil	KP256425
10	*P. nattereri*	NtrMS10	-	Rio Paraguay basin	Unknown	Brazil	KP256426
10	*P. nattereri*	NtrMS11	-	Rio Paraguay basin	Unknown	Brazil	KP256427
9	*P. nattereri*	NtrTO19	-	Rio Tocantins basin	Unknown	Brazil	KP256428
9	*P. nattereri*	NtrTO21	-	Rio Tocantins basin	Unknown	Brazil	KP256429
9	*P. nattereri*	NtrTO24	-	Rio Tocantins basin	Unknown	Brazil	KP256430
9	*P. nattereri*	NtrTO30	-	Rio Tocantins basin	Unknown	Brazil	KP256431
10	*P. nattereri*	OL-0485	-	Unknown	Unknown	Unknown	DSFRE372-08
10	*P. nattereri*	OL-0486	-	Unknown	Unknown	Unknown	DSFRE373-08
10	*P. nattereri*	OL-0487	-	Unknown	Unknown	Unknown	DSFRE374-08
6	*P. nattereri*	P1A	-	Introduced specimens	Metro Manila	Philippines	FCOD001-15
6	*P. nattereri*	P1B	-	Introduced specimens	Metro Manila	Philippines	FCOD002-15
6	*P. nattereri*	P1C	-	Introduced specimens	Metro Manila	Philippines	FCOD003-15
6	*P. nattereri*	P2C	-	Introduced specimens	Metro Manila	Philippines	FCOD006-15
6	*P. nattereri*	P3A	-	Introduced specimens	Metro Manila	Philippines	FCOD007-15
6	*P. nattereri*	P3B	-	Introduced specimens	Metro Manila	Philippines	FCOD008-15
6	*P. nattereri*	P3C	-	Introduced specimens	Metro Manila	Philippines	FCOD009-15
6	*P. nattereri*	NC_015840	-	Unknown	Unknown	Unknown	NC015840
8	*P. nattereri*	UEMA 104549	-	Rio Itapecuru basin	Rosário, Maranhão	Brazil	ITAPE137-15
8	*P. nattereri*	UEMA 104549	-	Rio Itapecuru basin	Rosário, Maranhão	Brazil	ITAPE138-15
8	*P. nattereri*	UEMA 104549	-	Rio Itapecuru basin	Rosário, Maranhão	Brazil	ITAPE139-15
8	*P. nattereri*	UEMA 104549	-	Rio Itapecuru basin	Rosário, Maranhão	Brazil	ITAPE140-15
8	*P. nattereri*	UEMA 104549	-	Rio Itapecuru basin	Rosário, Maranhão	Brazil	ITAPE141-15
8	*P. nattereri*	UEMA 104549	-	Rio Itapecuru basin	Rosário, Maranhão	Brazil	ITAPE142-15
8	*P. nattereri*	UEMA 104549	-	Rio Itapecuru basin	Itapecuru Mirim, Maranhão	Brazil	ITAPE144-15
8	*P. nattereri*	UEMA 104549	-	Rio Itapecuru basin	Itapecuru Mirim, Maranhão	Brazil	ITAPE145-15
8	*P. nattereri*	UEMA 104549	-	Rio Itapecuru basin	Itapecuru Mirim, Maranhão	Brazil	ITAPE146-15
8	*P. nattereri*	UEMA 104550	-	Rio Itapecuru basin	Mirador, Maranhão	Brazil	ITAPE148-15
8	*P. nattereri*	UEMA 104550	-	Rio Itapecuru basin	Mirador, Maranhão	Brazil	ITAPE149-15
8	*P. nattereri*	UEMA 104550	-	Rio Itapecuru basin	Mirador, Maranhão	Brazil	ITAPE150-15
6	*P. nattereri*	UFAM 643	643	Rio Amazonas basin	Pará	Brazil	MG752532
6	*P. nattereri*	UFAM 644	644	Rio Amazonas basin	Pará	Brazil	MG752533
9	*P. nattereri*	UFAM 2931	2931	Rio Araguaia basin	Pará	Brazil	MG752534
9	*P. nattereri*	UFAM 2932	2932	Rio Araguaia basin	Pará	Brazil	MG752535
9	*P. nattereri*	UFAM 2933	2933	Rio Araguaia basin	Pará	Brazil	MG752536
6	*P. nattereri*	UFAM 3579	3579	Rio Amazonas basin	Amazonas	Brazil	MG752537
6	*P. nattereri*	UFAM 3580	3580	Rio Amazonas basin	Amazonas	Brazil	MG752538
6	*P. nattereri*	UFAM 3581	3581	Rio Amazonas basin	Amazonas	Brazil	MG752539
6	*P. nattereri*	UFAM 3829	3829	Rio Paru, Amazon basin	Pará	Brazil	MG752542
6	*P. nattereri*	UFAM 3830	3830	Rio Paru, Amazon basin	Pará	Brazil	MG752543
6	*P. nattereri*	UFAM 3831	3831	Rio Paru, Amazon basin	Pará	Brazil	MG752544
9	*P. nattereri*	UFAM 3903	3903	Rio Tocantins basin	Pará	Brazil	MG752545
9	*P. nattereri*	UFAM 3904	3904	Rio Tocantins basin	Pará	Brazil	MG752546
9	*P. nattereri*	UFAM 3905	3905	Rio Tocantins basin	Pará	Brazil	MG752547
9	*P. nattereri*	UFAM 4556	4556	Rio Tocantins basin	Pará	Brazil	MG752548
9	*P. nattereri*	UFAM 4557	4557	Rio Tocantins basin	Pará	Brazil	MG752549
9	*P. nattereri*	UFAM 4558	4558	Rio Tocantins basin	Pará	Brazil	MG752550
6	*P. nattereri*	UFAM 5553	5553	Rio Xingu, Amazon basin	Pará	Brazil	MG752551
6	*P. nattereri*	UFAM 11497	11497	Rio Tapajós, Amazon basin	Pará	Brazil	MG752553
6	*P. nattereri*	UFAM 11498	11498	Rio Tapajós, Amazon basin	Pará	Brazil	MG752554
6	*P. nattereri*	UFAM 11500	11500	Rio Tapajós, Amazon basin	Pará	Brazil	MG752556
6	*P. nattereri*	UFAM 11501	11501	Rio Tapajós, Amazon basin	Pará	Brazil	MG752557
6	*P. nattereri*	UFAM 12603	12603	Rio Branco, Amazon basin	Roraima	Brazil	MG752558
6	*P. nattereri*	UFAM 12626	12626	Rio Branco, Amazon basin	Roraima	Brazil	MG752559
6	*P. nattereri*	UFAM 14105	14105	Rio Branco, Amazon basin	Roraima	Brazil	MG752560
6	*P. nattereri*	UFAM 14106	14106	Rio Branco, Amazon basin	Roraima	Brazil	MG752561
6	*P. nattereri*	UFAM 14107	14107	Rio Branco, Amazon basin	Roraima	Brazil	MG752562
6	*P. nattereri*	UFAM 14108	14108	Rio Branco, Amazon basin	Roraima	Brazil	MG752563
6	*P. nattereri*	UFAM 15261	15261	Rio Madeira, Amazon basin	Amazonas	Brazil	MG752564
6	*P. nattereri*	UFAM 15262	15262	Rio Madeira, Amazon basin	Amazonas	Brazil	MG752565
6	*P. nattereri*	UFAM 15263	15263	Rio Madeira, Amazon basin	Amazonas	Brazil	MG752566
11	*P. nattereri*	UFAM 15272	15272	Rio Guaporé, Amazon basin	Rondônia	Brazil	MG752567
11	*P. nattereri*	UFAM 15273	15273	Rio Guaporé, Amazon basin	Rondônia	Brazil	MG752568
11	*P. nattereri*	UFAM 15274	15274	Rio Guaporé, Amazon basin	Rondônia	Brazil	MG752569
11	*P. nattereri*	UFAM 15276	15276	Rio Guaporé, Amazon basin	Rondônia	Brazil	MG752570
11	*P. nattereri*	UFAM 15278	15278	Rio Guaporé, Amazon basin	Rondônia	Brazil	MG752572
4	*P. piraya*	INPA 56741	15283	Rio São Francisco basin	Minas Gerais/Bahia	Brazil	MG752583
4	*P. piraya*	INPA 56741	15284	Rio São Francisco basin	Minas Gerais/Bahia	Brazil	MG752584
4	*P. piraya*	INPA 56741	15285	Rio São Francisco basin	Minas Gerais/Bahia	Brazil	MG752585
3	*P. piraya*	LBP 11286	48749	Rio São Francisco basin	Gararu, Sergipe	Brazil	PYGO037-18
4	*P. piraya*	LBP 11286	48750	Rio São Francisco basin	Gararu, Sergipe	Brazil	PYGO038-18
3	*P. piraya*	LBP 11286	48751	Rio São Francisco basin	Gararu, Sergipe	Brazil	PYGO039-18
5	*P. piraya*	LBP 11300	42931	Rio São Francisco basin	S. Roque Minas, Minas Gerais	Brazil	PYGO046-18
7	*P. piraya*	LBP 11336	45522	Rio São Francisco basin	S. Roque Minas, Minas Gerais	Brazil	PYGO047-18
3	*P. piraya*	LBP 11337	45546	Rio São Francisco basin	Pirapora, Minas Gerais	Brazil	PYGO055-19
4	*P. piraya*	LBP 11337	45547	Rio São Francisco basin	Pirapora, Minas Gerais	Brazil	PYGO057-19
4	*P. piraya*	LBP 11337	45548	Rio São Francisco basin	Pirapora, Minas Gerais	Brazil	PYGO056-19
3	*P. piraya*	LBP 21613	47336	Rio São Francisco basin	Pirapora, Minas Gerais	Brazil	PYGO040-18
3	*P. piraya*	LBP 21613	47337	Rio São Francisco basin	Pirapora, Minas Gerais	Brazil	PYGO041-18
4	*P. piraya*	LBP 21613	47338	Rio São Francisco basin	Pirapora, Minas Gerais	Brazil	PYGO042-18
3	*P. piraya*	LBP 21613	47339	Rio São Francisco basin	Pirapora, Minas Gerais	Brazil	PYGO043-18
3	*P. piraya*	LBP 21612	59752	Rio São Francisco basin	Jenipatuba, Minas Gerais	Brazil	PYGO044-18
4	*P. piraya*	LBP 21612	59753	Rio São Francisco basin	Jenipatuba, Minas Gerais	Brazil	PYGO045-18
3	*P. piraya*	DCC502	-	Rio Pandeiros, São Francisco basin	Januária, Minas Gerais	Brazil	HQ600843
4	*P. piraya*	DCC503	-	Rio Pandeiros, São Francisco basin	Januária, Minas Gerais	Brazil	HQ600844
4	*P. piraya*	DCC499	-	Rio Pandeiros, São Francisco basin	Januária, Minas Gerais	Brazil	HQ600845
3	*P. piraya*	DCC501	-	Rio Pandeiros, São Francisco basin	Januária, Minas Gerais	Brazil	HQ600846
3	*P. piraya*	DCC532	-	Rio Pandeiros, São Francisco basin	Januária, Minas Gerais	Brazil	HQ600847
4	*P. piraya*	DCC500	-	Rio Pandeiros, São Francisco basin	Januária, Minas Gerais	Brazil	HQ600848
3	*P. piraya*	DCC306	-	Rio Pandeiros, São Francisco basin	Januária, Minas Gerais	Brazil	HQ600849
5	*P. piraya*	DCC1043	-	Rio São Francisco basin	Várzea da Palma, Minas Gerais	Brazil	HM405211
4	*P. piraya*	PrySF499	-	Rio São Francisco basin	Unknown	Brazil	KP256432
4	*P. piraya*	PrySF500	-	Rio São Francisco basin	Unknown	Brazil	KP256433
3	*P. piraya*	PrySF501	-	Rio São Francisco basin	Unknown	Brazil	KP256434
4	*P. piraya*	PrySF503	-	Rio São Francisco basin	Unknown	Brazil	KP256435
5	*P. piraya*	-	-	Unknown	Unknown	Unknown	DSFRE115-08
7	*P. piraya*	-	-	Unknown	Unknown	Unknown	DSFRE116-08
4	*P. piraya*	-	-	Unknown	Unknown	Unknown	DSFRE117-08
3	*P. piraya*	-	-	Unknown	Unknown	Unknown	DSFRE118-08
3	*P. piraya*	-	-	Unknown	Unknown	Unknown	DSFRE351-08
5	*P. piraya*	-	-	Unknown	Unknown	Unknown	DSFRE352-08
5	*P. piraya*	-	-	Unknown	Unknown	Unknown	DSFRE387-08
5	*P. piraya*	-	-	Unknown	Unknown	Unknown	DSFRE388-08
4	*P. piraya*	UFAM 15286	15286	Rio São Francisco basin	Minas Gerais/Bahia	Brazil	MG752586
3	*P. piraya*	UFAM 15287	15287	Rio São Francisco basin	Minas Gerais/Bahia	Brazil	MG752587
4	*P. piraya*	UFAM 15288	15288	Rio São Francisco basin	Minas Gerais/Bahia	Brazil	MG752588
3	*P. piraya*	UFAM 15289	15289	Rio São Francisco basin	Minas Gerais/Bahia	Brazil	MG752589
12	*S. elongatus*	UFAM 15260	15260	Rio Madeira basin	Amazonas	Brazil	MG752622
