# Supplementary figures and images for: Molecular Data Reveal Multiple Lineages in Piranhas of the Genus Pygocentrus (Teleostei, Characiformes)
Source: Genes (Basel). 2019 May 15;10(5):371. doi: 10.3390/genes10050371 (PMC6562675; doi:10.3390/genes10050371)

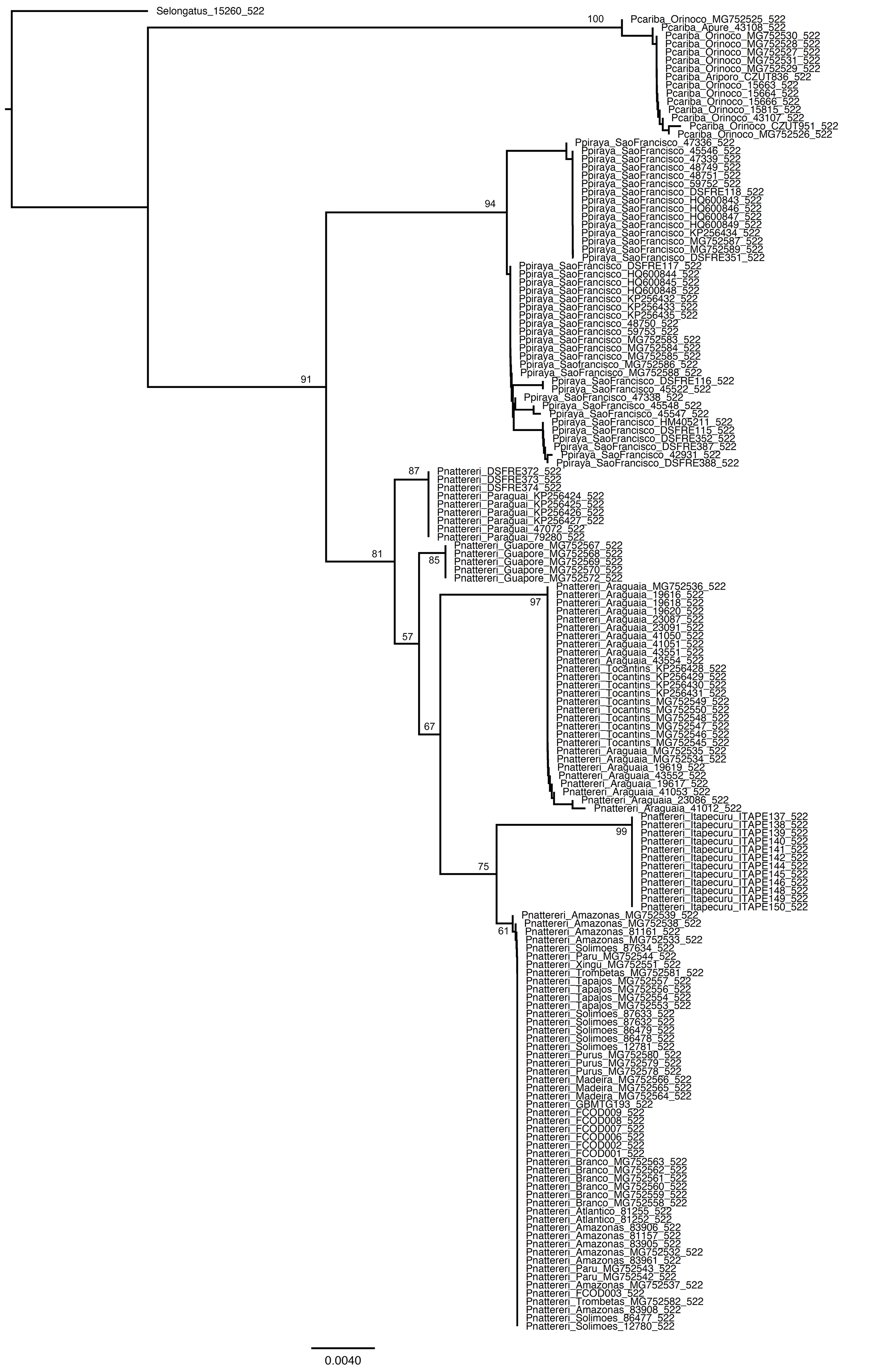

Supplement: Supplementary file 1 [file genes-10-00371-s001.zip › Supplementary/Fig S1 NJ.tif]

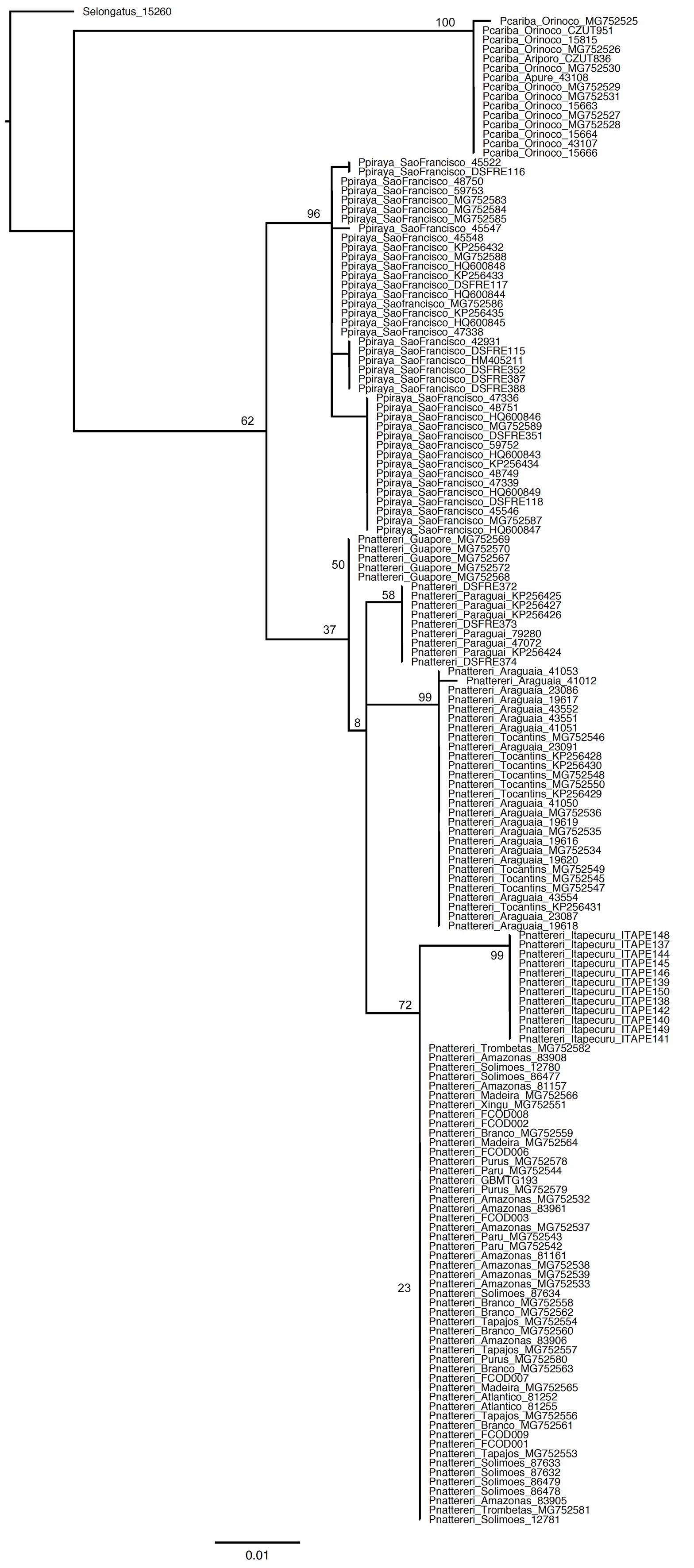

Supplement: Supplementary file 1 [file genes-10-00371-s001.zip › Supplementary/Fig S2 ML.tif]

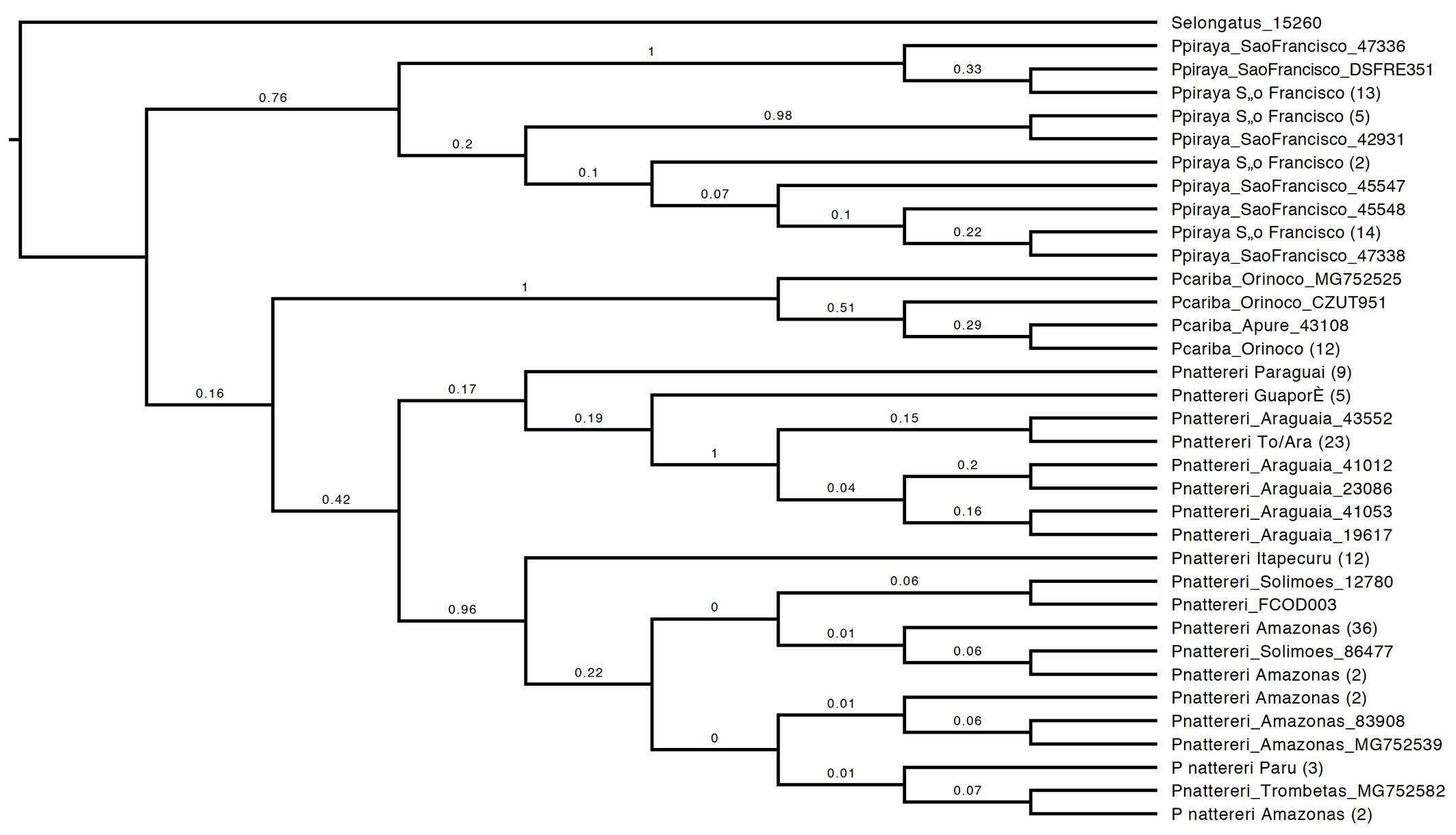

Supplement: Supplementary file 1 [file genes-10-00371-s001.zip › Supplementary/Fig S3 BI.tif]
